# Application of Multimodal Transformer Model in Intelligent Agricultural Disease Detection and Question-Answering Systems

**DOI:** 10.3390/plants13070972

**Published:** 2024-03-28

**Authors:** Yuchun Lu, Xiaoyi Lu, Liping Zheng, Min Sun, Siyu Chen, Baiyan Chen, Tong Wang, Jiming Yang, Chunli Lv

**Affiliations:** China Agricultural University, Beijing 100083, China

**Keywords:** agricultural large model, deep learning, smart agriculture, transformer model, agricultural disease detection

## Abstract

In this study, an innovative approach based on multimodal data and the transformer model was proposed to address challenges in agricultural disease detection and question-answering systems. This method effectively integrates image, text, and sensor data, utilizing deep learning technologies to profoundly analyze and process complex agriculture-related issues. The study achieved technical breakthroughs and provides new perspectives and tools for the development of intelligent agriculture. In the task of agricultural disease detection, the proposed method demonstrated outstanding performance, achieving a precision, recall, and accuracy of 0.95, 0.92, and 0.94, respectively, significantly outperforming the other conventional deep learning models. These results indicate the method’s effectiveness in identifying and accurately classifying various agricultural diseases, particularly excelling in handling subtle features and complex data. In the task of generating descriptive text from agricultural images, the method also exhibited impressive performance, with a precision, recall, and accuracy of 0.92, 0.88, and 0.91, respectively. This demonstrates that the method can not only deeply understand the content of agricultural images but also generate accurate and rich descriptive texts. The object detection experiment further validated the effectiveness of our approach, where the method achieved a precision, recall, and accuracy of 0.96, 0.91, and 0.94. This achievement highlights the method’s capability for accurately locating and identifying agricultural targets, especially in complex environments. Overall, the approach in this study not only demonstrated exceptional performance in multiple tasks such as agricultural disease detection, image captioning, and object detection but also showcased the immense potential of multimodal data and deep learning technologies in the application of intelligent agriculture.

## 1. Introduction

The rapid development of information technology and artificial intelligence has become a significant driving force in advancing modern agriculture [1], particularly in plant disease detection and management, where technological innovation and applications are key to ensuring agricultural production efficiency and food safety [2,3]. Traditional methods of plant disease detection, reliant on the experience and judgment of agricultural experts [4], are not only time-consuming and labor-intensive, but their accuracy and efficiency are limited by the constraints of the experts’ knowledge and experience [5].

The detection of plant fungal pathogens, discussed by Ray, Monalisa et al. [6], necessitates expertise in microbiology and is invariably influenced by individual experience. Vadamalai Ganesan et al. [7], employed plant genetics and physiology for disease detection, analyzing the impact of pathogens on host plants using proteomics; however, the accuracy of their method could not be guaranteed. To enhance precision, Das Debasish et al. [8] utilized various feature extraction techniques to classify different types of leaf diseases. They experimented with support vector machine (SVM), random forest, and logistic regression methods, finding SVM to be the most effective. However, their model, limited to binary classification of tomato leaves as healthy or diseased, failed to meet practical needs.

In response to these challenges, the urgency of incorporating intelligent technologies for accurate and rapid detection of plant diseases is evident [9]. In this context, this study introduces a disease detection and agricultural question-answering system based on multimodal and large language model technologies [10], aimed at enhancing the level of agricultural production intelligence and providing effective decision support for agricultural workers.

Several researchers have made significant contributions. For instance, Deepalakshmi P et al. [11] used CNN to extract features from input images to identify the diseased and healthy leaves of different plants, with their model taking an average of 3.8 s for disease detection and achieving a 94.5% accuracy rate. Sharma, Parul et al. [12] applied CNN to plant disease detection, reaching a 98.6% accuracy rate, though their method could fail in areas with multiple disease symptoms. Bedi Punam et al. [13] used a hybrid model of a convolutional autoencoder (CAE) network and CNN for peach tree disease detection, achieving a 98.38% accuracy rate in testing, but the small dataset size limited the model’s robustness. Given the potential loss of important information with CNN models, De Silva Malithi et al. [14] combined a CNN with ViT, achieving an 83.3% accuracy rate. To enhance accuracy, Parez Sana et al. [15] proposed the green vision transformer technique, employing ViT to reduce model parameters and improve accuracy, demonstrating real-time processing capability. Thai Huy-Tan et al. [16] designed the FormerLeaf model based on ViT for plant disease detection. They also proposed the LeIAP and SPMM algorithms for model optimization. Their experimental results showed a 15% improvement in inference speed, but they noted reduced model accuracy for complex background images and the dataset used in the experiments was unbalanced.

This study employs advanced computer vision models such as convolutional neural networks (CNN) [17] and YOLO (you only look once) [18], along with large language models like GPT [19] and BERT [20], to effectively detect plant diseases and accurately answer agriculture-related questions. The core of this research lies in proposing and implementing an innovative multimodal data processing approach and corresponding system architecture. A multi-transformer-based architecture was designed, capable of efficiently processing and integrating different modalities of data, such as images, text, and knowledge graphs, thereby achieving higher accuracy and efficiency in the identification and classification of plant diseases compared to traditional methods. This is of significant importance for the rapid identification and handling of agricultural diseases and for reducing crop losses. Moreover, a specialized question-answering system for the agricultural domain was constructed, combining large language models and expert knowledge graphs to understand complex agricultural questions and provide accurate, fact- and data-based answers. To train and validate our models, a comprehensive multimodal dataset, including rich image and textual data, was collected and constructed. This not only provided strong support for this study but also offers valuable resources for future research in related fields.

## 2. Related Works

### 2.1. Application of Multimodal Data in Agriculture

Recent developments in multimodal technologies have seen widespread application in the agricultural field, especially in disease detection and agricultural question-answering systems [21]. Multimodal technology refers to the integration and analysis of data from various modalities, such as images, text, and sound. In agriculture, this primarily involves a combination of image and text data. Image data typically originate from field photographs or satellite imagery, while text data may consist of professional literature or agricultural databases detailing crop cultivation and disease descriptions. Structurally, multimodal models generally encompass two main components: feature extraction from different modalities and multimodal fusion. For image data, convolutional neural networks (CNN) [22,23] or more advanced models like YOLO [24,25] are commonly used for spatial feature extraction. Text data, on the other hand, are processed using natural language processing techniques, such as the transformer model [26], to extract semantic features. Following feature extraction, multimodal fusion technology effectively combines the features from different modalities to facilitate more accurate classification, prediction, or generation. Matrix-based methods are a core technology in this process, involving the mathematical fusion of data from different modalities. Matrix factorization is a common technique in multimodal fusion that decomposes feature matrices from each modality to uncover shared latent features. Assuming the presence of data in two modalities, represented by matrices $X_{1}$ and $X_{2}$, matrix factorization aims to identify two low-rank matrices $U_{1}$, $U_{2}$ and a shared latent feature matrix *V*, satisfying the relation:

(1) $$X_{1}\approx U_{1}V,\;X_{2}\approx U_{2}V$$

Here, $U_{1}$ and $U_{2}$ represent the feature spaces of the two modalities, while *V* is the shared latent feature representation. Another method, canonical correlation analysis (CCA), seeks to maximize the correlation between feature vectors of two modalities. Given features *X* and *Y* from two modalities, CCA aims to find vectors $w_{x}$ and $w_{y}$ that maximize the projection correlation between *X* and *Y*:

(2) $$\max_{w_{x},w_{y}}\frac{w_{x}^{T}XY^{T}w_{y}}{\sqrt{w_{x}^{T}XX^{T}w_{x}}\sqrt{w_{y}^{T}YY^{T}w_{y}}}$$

Here, the terms $w_{x}^{T}XX^{T}w_{x}$ and $w_{y}^{T}YY^{T}w_{y}$ are included for normalization, ensuring that the results are not influenced by data scale. Joint Factor Analysis (JFA) is a matrix decomposition technique that simultaneously analyzes multiple data sources. Assuming *n* modalities with data matrices $X_{1},X_{2},\ldots ,X_{n}$, JFA aims to find a set of factor matrices $U_{1},U_{2},\ldots ,U_{n}$ and a shared latent feature matrix *V*, such that

(3) $$X_{i}\approx U_{i}V,\;\forall i\in 1,2,\ldots ,n$$

In this expression, each $U_{i}$ represents the feature space of the *i*th modality, while *V* is the cross-modal shared feature representation.

In applications such as disease detection in rice, wheat, potatoes, and cotton, multimodal technologies play a significant role [27,28]. For instance, in rice disease detection, a combination of field image data and literature descriptions of diseases [29] enables multimodal models to more accurately identify and classify different types of diseases. This not only enhances the precision of disease detection but also assists farmers in taking timely and effective measures to reduce losses. For agricultural question-answering systems, multimodal technologies have also demonstrated their robust capabilities. By integrating image recognition and natural language processing, such systems can provide more accurate and comprehensive answers. For example, farmers can upload images of crops and inquire about diseases. The system, by analyzing the images and consulting relevant agricultural knowledge bases, can provide specific disease information and prevention recommendations. Additionally, the unique advantage of multimodal technologies is exhibited when handling complex data. In agriculture, where environmental conditions are diverse and complex, a single modality often fails to provide sufficient information for accurate judgment [30]. Multimodal technology, by combining different types of data, offers a more comprehensive perspective, enhancing the model’s generalization capabilities and robustness. In practical applications, the challenges faced by multimodal technology include effectively integrating data from different modalities and designing universal models adaptable to various crops and disease types.

### 2.2. Application of Large Language Models in Agriculture

**Figure 1:**
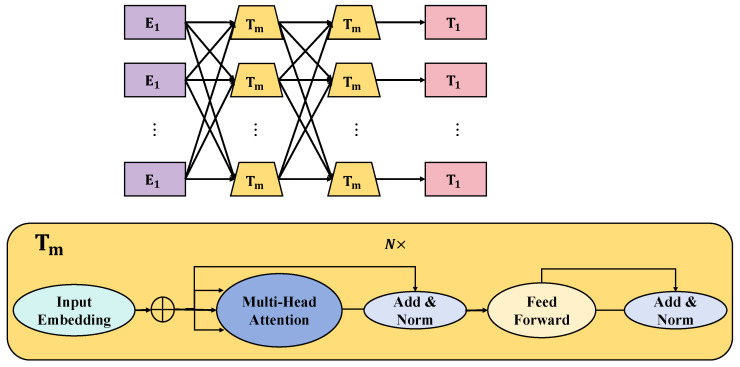
Structural diagram of the BERT model, demonstrating how input passes through an embedding layer and is processed through a multi-layer transformer network structure. This includes multi-head attention mechanisms, feedforward neural networks, and the addition of positional encoding.

Large language models, such as GPT and BERT [20], have made significant strides in various fields, including applications in agricultural disease detection and question-answering systems [31,32]. These models are renowned for their powerful semantic understanding and generation capabilities, providing effective tools for natural language processing. First, the structural features of large language models warrant attention. These models are typically based on deep learning, particularly the transformer architecture [33], learning rich language representations and knowledge through extensive data pre-training. Within the model, the multi-layer transformer network effectively captures long-distance dependencies in text through self-attention mechanisms, enabling the understanding and generation of complex language structures. The key components of the transformer are as follows: Self-attention, the core of the transformer, allows the model to focus on different positions in the input sequence. For a given input sequence, the self-attention mechanism calculates attention scores for each element with respect to the other elements in the sequence, as shown in Figure 1.

This can be expressed as [34]

(4) $$\mathrm{Attention}\left(Q,K,V\right)=\mathrm{softmax}\left(\frac{QK^{T}}{\sqrt{d_{k}}}\right)V$$

Here, *Q*, *K*, and *V* represent the query, key, and value matrices, respectively, derived from the input matrix through different weight matrix transformations. $\sqrt{d_{k}}$ is a scaling factor used to prevent excessively large values after the dot product. To enable the model to focus on information from different subspaces simultaneously, the transformer introduces a multi-head attention mechanism [34]. In this mechanism, the attention operation is divided into multiple heads, each independently calculating attention scores, which are then concatenated. This can be expressed as

(5) $$\mathrm{MultiHead}\left(Q,K,V\right)=\mathrm{Concat}\left({\mathrm{head}}_{1},{\mathrm{head}}_{2},\ldots ,{\mathrm{head}}_{h}\right)W^{O}$$

Here, each $\mathrm{head}*i=\mathrm{Attention}(QW_{i}^{Q},KW_{i}^{K},VW_{i}^{V})$ represents an independent attention mechanism, with $W_{i}^{Q}$, $W_{i}^{K}$, $W_{i}^{V}$, and $W^{O}$ being the parameters learned by the model. As transformers inherently lack a sequential order processing capability like RNNs, positional encoding is added to provide position information for elements in the sequence [34]. Positional encoding is usually a learnable parameter, added to the input sequence embedding, furnishing the model with positional information [34]. A common form of positional encoding is

(6) $$\mathrm{PE}\,\left(pos,2i\right)=\sin\left(pos/{10,000}^{2i/d\mathrm{model}}\right),\;\mathrm{PE}\,\left(pos,2i+1\right)=\cos\left(pos/{10,000}^{2i/d\mathrm{model}}\right)$$

Here, pos is the position, *i* is the dimension, and d∗model is the dimension of the model. Each encoder and decoder layer of the transformer contains a feed-forward network. This network, applying the same operations at each position [34], typically includes two linear transformations and an activation function, represented as

(7) $$\mathrm{FFN}\left(x\right)=\max\left(0,xW_{1}+b_{1}\right)W_{2}+b_{2}$$

Here, $W_{1}$, $W_{2}$, $b_{1}$, and $b_{2}$ are network parameters [34].

In specific applications such as disease detection in rice, wheat, potatoes, and cotton, as well as agricultural question-answering systems, the transformer model leverages its strong semantic understanding capabilities to analyze text information [34], such as disease descriptions and agricultural practice guidelines [35]. This analytical capability is crucial for enhancing the accuracy of disease diagnostics and answering agriculture-related questions. In the agricultural field, especially in disease detection and question-answering systems for crops like rice, wheat, potatoes, and cotton, the application of large language models is particularly important. For instance, in disease detection, the model can provide in-depth understanding and suggestions regarding diseases by analyzing agricultural text materials, such as disease descriptions and treatment methods [27]. Additionally, large language models can be combined with image recognition technology to provide more accurate disease diagnostics by analyzing images related to diseases and their descriptions. In agricultural question-answering systems, the role of large language models is indispensable. They can not only understand user queries but also generate information-rich, accurate responses. This is especially crucial for agriculture-related queries requiring expert knowledge. For example, farmers may inquire about methods for identifying or treating specific crop diseases, and large language models can provide professional and specific answers based on their extensive knowledge base [36].

### 2.3. Application of Computer Vision Techniques in Agriculture

**Figure 2:**
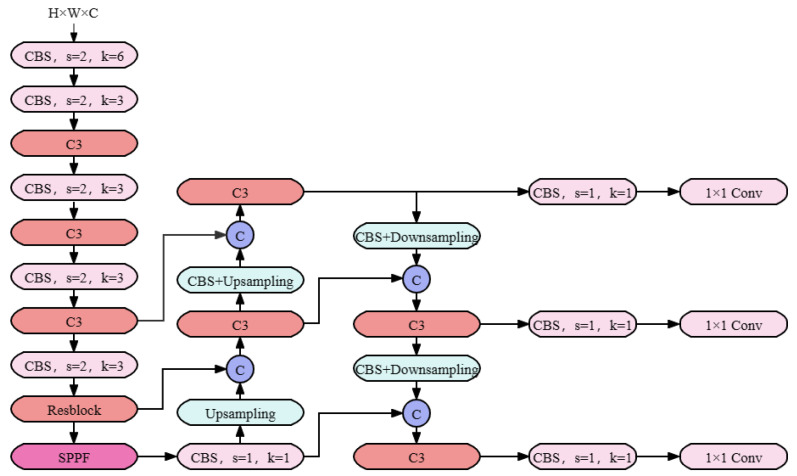
Structure diagram of the YOLOv5 object detection model, detailing the data flow from the input layer to the prediction layer, including input processing, backbone network, feature pyramid network (neck), and the different types of neural network modules used in each stage of prediction.

The application of computer vision models, particularly convolutional neural networks (CNN) and YOLO (you only look once), as shown in Figure 2, has been increasingly observed in the agricultural sector, especially in disease detection and agricultural question-answering systems [12,37].

CNNs, designed for processing data with a grid-like structure such as images, are deep neural networks centered around convolutional layers. These layers extract local features from images through convolution operations, which can be mathematically expressed as

(8) $$F_{ij}=\sum_{m}\sum_{n}I_{\left(i+m)(j+n\right)}K_{mn}$$

Here, *I* represents the input image, *K* the convolutional kernel, and $F_{ij}$ the convolutional output. This formula indicates that convolutional layers slide the kernel over the image, computing the dot product between the kernel and local regions of the image to extract features. In addition to convolutional layers, CNNs typically include activation layers and pooling layers. Activation layers, such as the ReLU function, introduce non-linearity, enabling the network to capture more complex features. Pooling layers, on the other hand, reduce the spatial dimensions of the features, enhancing the model’s generalization capabilities. A common pooling operation, max pooling, is mathematically expressed as

(9) $$P_{ij}=\max_{a,b\in [0,k-1]}F_{i+a,j+b}$$

Here, $P_{ij}$ denotes the pooling output, with *k* representing the size of the pooling window. The core advantage of these models lies in their ability to efficiently process and analyze large volumes of image data, thereby identifying specific patterns and objects. For instance, in the detection of diseases in crops like rice, wheat, potatoes, and cotton, CNNs initially perform feature extraction on the input crop images. Using these features, CNNs can identify different types of crop diseases. For example, in rice disease detection, extracted features might include the size, shape, and color of spots on the leaves [38].

YOLO is a popular single-stage object detection model that conceptualizes object detection as a regression problem. Unlike traditional step-by-step methods (such as first generating candidate regions and then classifying), YOLO directly predicts both the categories and positions of targets in a single network. In the YOLO model, the input image is divided into an S×S grid, with each grid cell responsible for predicting targets within that area. The output of YOLO can be represented as a vector, containing class probabilities, bounding box coordinates, and confidence scores. The mathematical representation of each bounding box is

(10) $$\left(box_{x},box_{y},box_{w},box_{h},C\right)$$

where $\left(box_{x},box_{y}\right)$ are the coordinates of the center of the bounding box, $\left(box_{w},box_{h}\right)$ its width and height, and *C* the confidence score of the bounding box containing a target. The loss function of the YOLO model, comprising category loss, localization loss, and confidence loss, is a crucial component. The loss function can be represented as

(11) $$\begin{array}{c} L=\lambda_{coord}\sum_{i=0}^{S^{2}}\sum_{j=0}^{B}l_{ij}^{obj}\left[{\left(x_{i}-\hat{x}*i\right)}^{2}+{\left(y_{i}-\hat{y}\,i\right)}^{2}\right]\\ +\lambda \,coord\sum *{i=0}^{S^{2}}\sum_{j=0}^{B}l_{ij}^{obj}\left[{\left(\sqrt{w_{i}}-\sqrt{\hat{w}*i}\right)}^{2}+{\left(\sqrt{h_{i}}-\sqrt{\hat{h}\,i}\right)}^{2}\right]\\ +\sum \,{i=0}^{S^{2}}l*i^{obj}{\left(C_{i}-\hat{C}*i\right)}^{2}+\lambda *noobj\sum_{i=0}^{S^{2}}l_{i}^{noobj}{\left(C_{i}-\hat{C}*i\right)}^{2}\\ +\sum *{i=0}^{S^{2}}\sum_{c\in classes}p_{i}\left(c\right)\log\left({\hat{p}}_{i}\left(c\right)\right) \end{array}$$

Here, $\lambda_{coord}$ and $\lambda_{noobj}$ are weight coefficients, $l_{ij}^{obj}$ indicates the presence of a target, $\left(x_{i},y_{i},w_{i},h_{i}\right)$ are the predicted bounding box parameters, $\left({\hat{x}}_{i},{\hat{y}}_{i},{\hat{w}}_{i},{\hat{h}}_{i}\right)$ the actual bounding box parameters, $C_{i}$ the predicted confidence score, ${\hat{C}}_{i}$ the actual confidence score, and $p_{i}\left(c\right)$ the probability of class *c*. The YOLO model excels in real-time disease detection, swiftly locating and classifying diseases within images. In cotton disease detection, YOLO can rapidly identify affected areas, assisting farmers in timely interventions [39]. Agricultural question-answering systems can utilize CNN or YOLO models to analyze crop images uploaded by users, then combine the analysis results with historical data to provide professional advice. Initially, the system analyzes uploaded wheat leaf images through visual models, subsequently integrating the analysis with agricultural knowledge bases to suggest possible disease causes and recommended treatment methods.

## 3. Results and Discussion

### 3.1. Disease Detection Results

The primary aim of this experiment was to compare and analyze the performance of various deep learning models in agricultural disease detection tasks, including AlexNet [40], GoogLeNet [41], VGG [22], ResNet [23], and the method proposed in this study. The experimental results are presented using the evaluation metrics of precision, recall, and accuracy to showcase the performance of each model. The experimental results are shown in Table 1.

As an early landmark model in deep learning, AlexNet has a relatively simple structure, comprising five convolutional layers and three fully connected layers. Although it achieved significant breakthroughs in early image processing tasks, its performance is relatively weaker when handling more complex agricultural disease detection tasks. This is primarily due to the limited feature extraction capability of AlexNet, especially in capturing subtle features, such as early signs of disease. Consequently, AlexNet showed the most modest performance in terms of precision, recall, and accuracy. GoogLeNet, introducing the Inception module, uses convolutional kernels of different sizes in the same layer, enabling it to capture features at different scales. This design makes GoogLeNet more powerful for feature extraction than AlexNet, especially in processing agricultural images with multi-scale features. Therefore, in the experiment, GoogLeNet’s performance showed an improvement, but it still had limitations in handling extremely complex agricultural data, due to its relatively simple network structure. VGG significantly enhances the model’s feature extraction capability with a deeper network structure (up to 19 layers) and small convolutional kernels. In agricultural disease detection tasks, VGG can better capture complex disease features, such as minute spots or discoloration. However, a major drawback of VGG is its bulky network structure with numerous parameters, leading to a lower computational efficiency in training and inference. ResNet solves the problem of vanishing gradients in deep networks by introducing residual connections, allowing the network to deepen (versions of ResNet reach up to 152 layers) without losing training efficiency. This combination of depth and residual structure enables ResNet to excel in capturing complex, hierarchical features. Thus, in agricultural disease detection tasks, ResNet significantly outperforms preceding models in terms of precision, recall, and accuracy. For example, the MAF-ResNet50 proposed in [4] enhances the model expressiveness of ResNet50 by designing parallel activation function layers to improve the accuracy of corn disease recognition. However, it could only achieve a recognition accuracy higher than 95% on four types of corn disease samples, which still lagged behind the generalization ability of the method proposed in this paper. The method proposed in this paper builds upon the foundation of these models with further innovations and optimizations. Specific details might include more complex network structures, more effective feature fusion mechanisms, and algorithms specifically optimized for agricultural disease detection tasks. These innovations enabled the proposed method to more effectively integrate information from different sources and capture more detailed disease features when processing the multimodal data. Consequently, in the experiment, the proposed method demonstrated the optimal performance for all evaluation metrics.

### 3.2. Agricultural Image Captioning Experiment Results

The design of this experiment aimed to evaluate and compare the performance of various deep learning models in the task of generating descriptive text from agricultural images. This task involved the automatic generation of descriptive text from agricultural images, which is significant for enhancing the level of automation and intelligence in agricultural management. By comparing the precision, recall, and accuracy of the different models, insights were gained into each model’s ability to understand and describe agricultural images. In the following experimental results, the performance of BLIP [42], mPLUG-Owl [43], InstructBLIP [44], CLIP [45], BLIP2 [46], and the method proposed in this paper on the agricultural image captioning task is demonstrated. The experimental results are shown in Table 2.

BLIP (bootstrap your own latent) is an earlier deep learning model with certain capabilities in handling the fusion of image and text, but due to its relatively simple network structure and training strategy, it exhibits average performance in complex agricultural image captioning tasks. This was reflected in its lower precision, recall, and accuracy. mPLUG-Owl, an improved version of the multimodal learning model, shows enhancements in processing image and language fusion, particularly in understanding image content and generating relevant text. However, due to limitations in feature extraction and associative learning, mPLUG-Owl’s performance in agricultural image captioning tasks remains limited. The InstructBLIP model introduces more advanced training strategies and network structures, particularly excelling in understanding image content and generating accurate descriptions in image and text fusion tasks. This improvement can be attributed to its enhanced feature extraction capability and text generation strategy, leading to significant improvements in performance for agricultural image captioning tasks. The CLIP (contrastive language-image pretraining) model is pre-trained on large-scale datasets through contrastive learning, strengthening the model’s ability to understand image content and related text. This training approach endows CLIP with advantages in understanding complex agricultural images and generating accurate descriptions, thereby performing well in all evaluation metrics. BLIP2, as an advanced version of BLIP, has had further optimization in network structure and training strategy. These improvements make BLIP2 more efficient in handling complex image and text fusion tasks, particularly excelling in understanding the details of agricultural images and generating precise descriptions.

### 3.3. Results for Object Detection

The object detection experiment conducted in this study was designed to evaluate and compare the performance of various deep learning models in agricultural disease detection tasks. This task holds significant importance for precision agriculture and intelligent agricultural management. By comparing the precision, recall, and accuracy of the different models, the performance of SSD [47], RetinaNet [48], CenterNet [49], YOLOv8 [50], and the method proposed in this paper in agricultural disease detection tasks was examined. The experimental results are displayed in Table 3 and Table 4.

SSD (single shot multibox detector) is a one-stage object detection model known for its speed and simplicity of implementation. SSD directly predicts the categories and locations of objects in a feature map, without the need for additional candidate region generation steps. However, due to its relatively simple network structure, SSD’s precision and recall are relatively lower when processing complex and small-scale targets, as reflected in its lower experimental scores. RetinaNet introduced focal loss to address the issue of class imbalance, which is particularly effective in scenarios with a large number of negative samples. Its performance improved compared to SSD, as evidenced by its increased precision and recall. However, the computational complexity of RetinaNet is relatively high, which may limit its practical application. CenterNet utilizes a keypoint-based object detection approach, detecting the center points of objects and regressing their sizes for target localization. This method is more direct and efficient compared to traditional bounding box prediction approaches. CenterNet outperformed both SSD and RetinaNet in precision and recall, indicating its better capability for locating small targets and handling complex scenes. The YOLO series, known for its speed and good performance, with YOLOv8 as its latest version, introduced multiple innovations in network structure and algorithms, further enhancing the model’s detection capabilities. YOLOv8 excelled in the agricultural disease detection task, with a high precision and recall demonstrating its excellent target localization and classification abilities. For instance, the YOLO-based object detection model discussed in [51] achieved a mAP of 0.6991 on the wheat dataset provided by Kaggle. This performance was significantly different from that of the method proposed in our paper. The reason for this was that the method in the cited paper only fine-tuned the loss function on the basis of a single-stage object detection network and did not leverage attention mechanisms or data from other modalities for model enhancement. On the other hand, our method, by integrating the latest deep learning technologies and specifically optimizing for the task of agricultural disease detection, achieved the best performance across all evaluation metrics. This was due to the more effective feature extraction mechanism, more refined target localization strategy, and more efficient classification algorithms. The high precision, recall, and accuracy of our method demonstrate its significant advantages in recognizing a variety of agricultural diseases.

### 3.4. Multimodal Dataset Ablation Experiment

In the multimodal dataset ablation experiment section of this paper, the goal was to explore the impact of different data modalities (image, text, sensor data) on the performance of the model. This experiment, by comparing the precision, recall, and accuracy of the model with different combinations of data modalities, aimed to reveal the contribution of the various data modalities to model performance and their interactions. The experimental results are presented in Table 5.

When the model used all modal data (image, text, and sensor data) simultaneously, it exhibited optimal performance, with a precision, recall, and accuracy of 0.96, 0.93, and 0.94, respectively. This indicates that the combination of these three data modalities provided the model with the richest and most comprehensive information, greatly enhancing the model’s accuracy in identifying and classifying diseases. Image data offer intuitive visual information, text data provide descriptive and background information, and sensor data contribute additional environmental and condition information. The integration of these data allows the model to more comprehensively understand and analyze agricultural diseases. When only sensor data were used, all performance indicators significantly decreased, with a precision, recall, and accuracy of 0.24, 0.21, and 0.23, respectively. This suggests that relying solely on sensor data is insufficient for complex agricultural disease detection tasks. In [52], this phenomenon was also reflected; the multimodal dataset proposed there demonstrated how the use of sensor data can enhance the application of artificial intelligence technologies in agricultural automation scenarios. Although sensor data can provide environmental condition information, they lack direct descriptive features of specific diseases, limiting the model’s performance in identifying specific diseases. When only text data were used, the model’s performance improved but was still inferior to the full modal data, with a precision, recall, and accuracy of 0.78, 0.73, and 0.75, respectively. This indicates that the text data aided the model by providing disease descriptions and background information but lacked the intuitiveness of image data and the environmental information of sensor data. When only image data were used, there was a significant improvement in performance, with a precision, recall, and accuracy of 0.92, 0.90, and 0.91, respectively. This demonstrates the crucial role of image data in agricultural disease detection, where visual information is highly effective for disease identification and classification. However, without the assistance of text and sensor data, the model still lacked comprehensiveness and contextual understanding of the disease. The results of the multimodal dataset ablation experiment lead to the conclusion that different data modalities contribute differently to agricultural disease detection tasks. Image data play a central role in providing intuitive visual information, text data contribute significantly in providing background and descriptive information, and sensor data offer valuable environmental and condition information. The integration of these data enabled the model to fully understand and accurately identify agricultural diseases, exhibiting outstanding performance. Therefore, the fusion of multimodal data is crucial for enhancing the accuracy of agricultural disease detection.

### 3.5. Different Loss Function Ablation Experiment

The ablation experiment on different loss functions in this study aimed to investigate the impact of various types of loss functions on the performance of tasks in disease detection, agricultural image captioning, and object detection. Loss functions play a crucial role in the training process of deep learning models, determining how the model evaluates the difference between predicted and actual results. Different types of loss functions may lead models to focus on different aspects, thereby affecting the final performance. The experiment examined three loss functions: hinge loss, mean squared error (MSE) loss, and multimodal loss, and their performance in three different tasks. The experimental results are presented in Table 6.

In the disease detection task, the multimodal loss achieved the best performance (precision: 0.95, recall: 0.92, accuracy: 0.94), followed by MSE Loss, with hinge loss performing the worst. Hinge loss, a loss function for classification tasks, aims to maximize the margin between correct and incorrect classifications. While effective in some classification tasks, it may not be sufficient for complex disease detection tasks, especially in cases involving multiple categories and subtle features. MSE Loss, which calculates the squared difference between predicted and actual values, is commonly used in regression tasks. In disease detection, it may capture subtle differences better than hinge loss, thereby improving model precision and recall. The multimodal loss, specifically designed for this study, considers the characteristics of different modal data, enabling the model to learn more effectively from multimodal data. This design resulted in the multimodal loss outperforming the other approaches in the disease detection task, reflecting its effectiveness in handling complex data. In the agricultural image captioning task, the multimodal loss also showed the best performance, followed by MSE loss, with hinge loss being the weakest. This result further confirms the effectiveness of multimodal loss in handling complex, diverse data. Agricultural image captioning involves not only image understanding but also the generation of semantically accurate descriptions, requiring the loss function to consider both the image content and the quality of text generation. In the object detection task, the multimodal loss outperformed the other two loss functions, demonstrating a precision of 0.96, recall of 0.92, and accuracy of 0.94. Object detection requires not only accurate identification of targets but also precise localization, demanding a loss function that can handle both aspects. The multimodal loss likely better balances these requirements, thereby improving the overall performance.

### 3.6. Limitations and Future Work

In this study, a comprehensive approach based on multimodal data and the transformer model was proposed to address key challenges in agricultural disease detection and question-answering systems. Despite the experimental results showing the excellent performance of our method across multiple tasks, there remain some limitations that require further improvement and expansion in future work.

Firstly, regarding data limitations. Although multimodal datasets, including image, text, and sensor data, were used in the experiments, the diversity and coverage of these data were still limited. For instance, in terms of the image data, while various disease images of multiple crops were collected, they might not have covered all types of crops and all possible disease conditions. Additionally, the text data mainly being sourced from existing agricultural literature and reports may have limited the model’s ability to handle non-standard texts or colloquial descriptions. As for the sensor data, the experiments primarily relied on data from specific environments, which may not have sufficiently represented the complexity and diversity of all agricultural environments. Second, regarding the model limitations. Although our multimodal transformer model excelled in processing multimodal data, there are still potential issues. For example, while the transformer model has advantages in processing long sequence data, its computational complexity is high, which may not be suitable for resource-limited environments. Additionally, although the multimodal alignment module can effectively integrate data from different modalities, its alignment mechanism may still need improvement to better handle heterogeneity and complex interactions between different modalities. In terms of the loss function, although the multimodal loss designed by us performed well in multiple tasks, its design and optimization require further research. Particularly in balancing the contributions of different modal data and adapting to the requirements of different tasks, more experiments and theoretical analysis might be needed for guidance.

Future work directions mainly include the following aspects. First, further expansion in data diversity and coverage is planned. More diverse agricultural data will be collected, including a wider range of crop types, data from different regions and environmental conditions, as well as richer textual descriptions and sensor data. This will help improve the model’s generalizability and practicality. Second, further optimization of model performance is planned. Future work will focus on reducing the computational complexity of the transformer model, to make it more suitable for resource-limited environments. Additionally, more efficient multimodal data alignment and fusion mechanisms will be explored to better handle heterogeneity and complex relationships between different modal data. In-depth research on multimodal loss is planned, including exploring the impact of different tasks on the loss function, how to better balance the contributions of different modal data, and how to adapt to the characteristics and requirements of different tasks. Efforts will also be made to apply the model in real agricultural environments for large-scale field testing and validation. This will help assess the practicality and effectiveness of the model and provide real feedback for further optimization.

## 4. Materials and Methods

### 4.1. Dataset Collection

#### 4.1.1. Corpus Construction

In the construction of the corpus for this study, the sources, quantity, and methods of data acquisition were first clarified. The primary goal was to collect text data encompassing a broad range of agricultural knowledge, especially concerning diseases related to crops such as rice, wheat, potatoes, and cotton. Sources included the databases of various agricultural research institutions, agricultural technology websites, professional forums, and scientific research paper libraries. National agricultural information platforms and the databases of agricultural science and technology journals were accessed. Through these channels, over one hundred thousand records were collected, covering topics such as crop cultivation, disease identification, treatment methods, and prevention strategies. The rationale for using these data was multifaceted. First, they encompass a wide range of information from basic agricultural knowledge to advanced technical expertise, crucial for building a comprehensive agricultural question-answering system. Second, the accuracy and professionalism of these data were ensured, as they originated from authoritative and reliable platforms. Lastly, the diversity in language expression and structure of these text data aided in enhancing the generalization ability of our model. In terms of data annotation, a semi-automated approach was adopted. Initially, natural language processing technology was used for data preprocessing, including tokenization, part-of-speech tagging, syntactic analysis, etc. These steps helped to better understand the text’s structure and semantics. Subsequently, key information in the data, such as crop names, disease types, and symptom descriptions, were automatically annotated using rule-based methods. This process can be represented by the following equation:

(12) T=Tag(W;ρ)

Here, *W* represents the preprocessed text words, *T* the annotated tags, and ρ the set of annotation rules. However, automatic annotation cannot fully replace the accuracy of manual annotation. Therefore, a professional annotation team was organized to review and correct the results of automatic annotation. In the corpus construction process, text vectorization technology was employed, converting text into a format processable by machine learning models. Specifically, word embedding techniques such as Word2Vec and BERT were used to transform each word in the text into a vector in a high-dimensional space. This process can be expressed as follows:

(13) V=Embed(W;η)

Here, *V* represents word vectors, *W* is the word, and η the parameters of the word embedding model. Through this method, semantic relationships and contextual information between words were captured, crucial for the subsequent model training and knowledge extraction. The final corpus not only contained a large amount of annotated text but was also converted into a format suitable for machine learning through word embedding technology. This corpus served as the foundational dataset for training our agricultural question-answering system and disease identification models, with its comprehensiveness and accuracy having a decisive impact on the improvement in system performance.

#### 4.1.2. Knowledge Graph Construction

**Figure 3:**
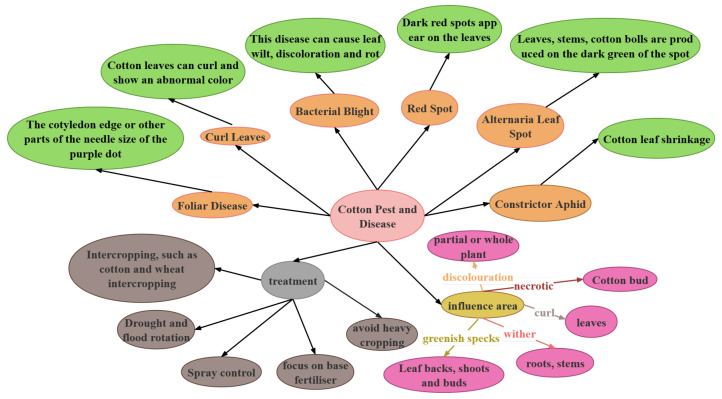
Knowledge graph of the relationship between cotton growth and diseases, showing typical symptoms during the cotton growth process, possible diseases, related pests, and corresponding treatment methods.

The construction of the knowledge graph was a core component of this study, involving meticulous and systematic work aimed at providing a solid and comprehensive knowledge base for the agricultural question-answering system and disease detection models. The knowledge graph construction process focused not only on collecting and organizing data but also on its in-depth processing and intelligent application. Initially, data sources included the aforementioned annotated corpus dataset, and additional data were gathered from agricultural technology forums and communities, involving actual questions and discussions about crop diseases by farmers. The goal was to collect over one million independent data records to form a comprehensive knowledge system, as shown in Figure 3.

During the data annotation process, a combination of natural language processing technology and artificial intelligence was employed. Text analysis tools automatically identified key entities and concepts in the text, such as disease names, symptom descriptions, and management methods. However, considering the specificity and complexity of the agricultural field, experts in agriculture were also invited for manual reviewing and supplementary annotation. This process ensured the accuracy and professionalism of the data annotation. The annotation process can be represented by the following equation:

(14) R=Annotate(E,β)

Here, *R* represents the relationships between entities, *E* the entities, and β the set of annotation rules. Using this method, information in the knowledge graph was ensured to be both accurate and comprehensive. Next came the construction process of the knowledge graph. After defining the types of entities and relationships, graph database technology was used to store and organize this information. This step, central to constructing the knowledge graph, involved not only data storage but also efficient organization and retrieval of these data. The construction process can be summarized by the following equation:

(15) G=BuildGraph(E,R)

Here, *G* represents the knowledge graph, and *E* and *R* respectively are the sets of entities and relationships. The aim was to build a knowledge graph that not only reflects actual agricultural knowledge but also supports efficient querying and analysis. Through the aforementioned steps, a knowledge graph covering a broad range of agricultural knowledge, with clear structure and dynamic updating, was constructed. This graph not only provided strong knowledge support for the agricultural question-answering system but also the necessary background information for the disease detection model. Its construction greatly enhanced the performance of these systems, enabling them to serve agricultural production and research more accurately and efficiently.

#### 4.1.3. Sensor Data Collection

In this study, the collection of sensor data was crucial for building a comprehensive agricultural knowledge graph and enhancing the accuracy of the disease detection system. The sensor data we collected originated from smart agricultural monitoring equipment, such as soil testing sensors and plant growth monitoring devices, which can provide detailed data on soil pH, electrical conductivity, nutrient content, and plant physiological indicators. These devices are deployed at key locations in fields and regularly collect data to monitor and assess crop growth condition and potential disease risks. All collected sensor data underwent strict data cleaning and preprocessing to ensure the data quality met the requirements for the subsequent analysis. During the data preprocessing process, we eliminated outliers, filled in missing values, and normalized the data to facilitate their use in later data analysis.

#### 4.1.4. Image Data Collection

In this study, the collection of image data was crucial for establishing an efficient disease detection model and enhancing the performance of the agricultural question-answering system. The content, sources, quantity, and methods of acquiring image data were fundamental to building accurate and comprehensive models. First, the content of the image data primarily included images of healthy and diseased plants of major crops such as rice, wheat, potatoes, and cotton, as shown in Table 7.

**Figure 4:**
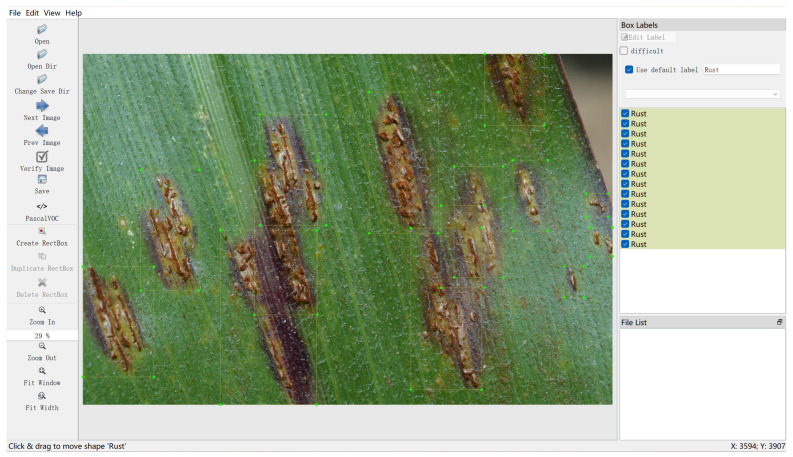
Screenshot of the dataset labeling interface, demonstrating the precise labeling of disease lesions on individual plant leaves in an agricultural disease detection dataset using annotation tools. This was carried out to create a labeled dataset for machine learning model training.

These images included different growth stages of the crops and manifestations of various common and rare diseases. For rice, images of healthy rice, rice affected by blast disease, yellow leaf disease, and other disease conditions were collected. For each type of crop, efforts were made to ensure that the images covered stages from early symptoms to severe infection. Additionally, the sources of the data were diverse, mainly collected from the West District Botanical Garden of China Agricultural University. Furthermore, a certain number of images were obtained from public resources on the internet, representing crops from different regions and under various climatic conditions. The rationale for using these image data was that a rich and diversified image dataset is key to building an efficient disease detection model. The diversity in crop types, disease types, and stages of disease development significantly enhanced the model’s generalization capability and accuracy. Moreover, images under different lighting conditions and shooting angles helped train the model to better adapt to various situations in practical applications. The annotation process involved identifying disease areas in the images and assigning correct disease category labels. For some complex images, expert knowledge was used for precise annotation, as shown in Figure 4.

### 4.2. Data Preprocessing

#### 4.2.1. Preprocessing of Corpus Data

**Figure 5:**
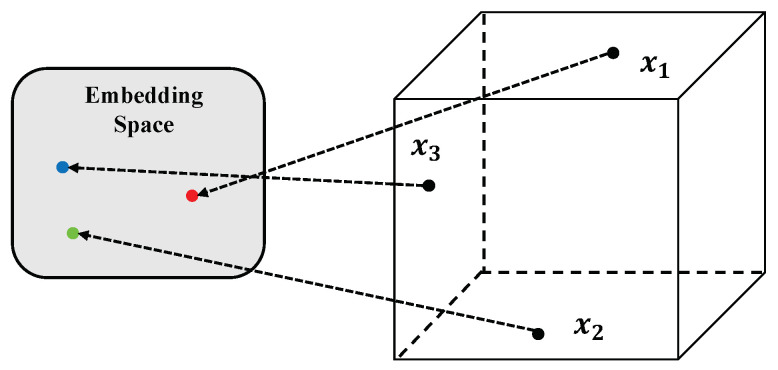
Schematic diagram of text embedding in a three-dimensional space, displaying how text data are mapped onto points in an embedding space formed by three base vectors $x_{1}$, $x_{2}$, and $x_{3}$.

In this study, the preprocessing of corpus data was a key step in building an efficient agricultural question-answering system and disease detection model. The preprocessing involved transforming raw text data into a format more amenable to computer processing for subsequent machine learning and natural language processing tasks. A series of preprocessing techniques were employed to optimize the corpus data, ensuring data quality and processing efficiency. Initially, the raw corpus data, sourced from various channels including agricultural papers, technical reports, online forums, and Q&A, exhibited significant structural and format differences. To enable effective processing by the machine learning models, basic data cleaning was performed. This included removing irrelevant information (such as advertisements, meaningless characters), standardizing data formats (like dates, units), and correcting obvious errors. Subsequently, the text data underwent tokenization. Tokenization is the process of splitting long sentences or paragraphs in the text into individual words, which is particularly crucial for Chinese texts, due to the absence of clear delimiters between words. Efficient tokenization was carried out using statistical and machine-learning-based tools. In this paper, the text serialization operation utilized the existing tokenization tool Jieba for word segmentation, along with the Word2Vec model. Furthermore, the text data were subjected to part-of-speech tagging and syntactic analysis. Part-of-speech tagging involves assigning a grammatical role (such as noun, verb, etc.) to each word in the text, while syntactic analysis explores the dependency relationships between words in a sentence. These steps were vital for understanding the semantic structure of the text. To enhance the model performance and accuracy, the text data were further subjected to vectorization. Text vectorization involved converting words in the text into numerical vectors processable by computers. Word embedding technology [20] was employed for this transformation. Word embedding technology captures semantic relationships between words and translates these into vectors in a high-dimensional space, as shown in Figure 5.

The word vectorization process can be represented as

(16) V=Embed(W;η)

Here, *V* represents the word vectors, Embed is the word embedding function, and η indicates the parameters of the word embedding model. This step was crucial, as it directly impacted the performance of the subsequent models. Through these preprocessing steps, raw text data were transformed into a format suitable for machine learning and natural language processing. These preprocessing techniques not only enhanced the quality and consistency of the data but also laid a solid foundation for subsequent model training and analysis.

#### 4.2.2. Preprocessing of Image Data

**Figure 6:**
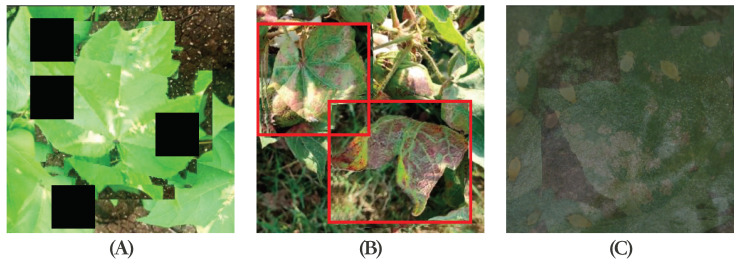
Example of the application of image enhancement techniques in agricultural disease detection: (**A**) The image shows plant images enhanced using the Cutout technique, (**B**) the image displays plant images enhanced using the Cutmix technique (red boxes mean the adding parts), (**C**) the image showcases plant images enhanced with color and brightness adjustments.

This paper discusses three methods of image data preprocessing: basic augmentation, Cutout, and Cutmix, as shown in Figure 6.

These methods are widely used to enhance image data, improving the performance of deep learning models. Cutout [53] is an image data augmentation technique that introduces randomness by obscuring part of the image, thereby mitigating overfitting and enhancing the model’s generalizability. The concept involves drawing a black square at a random location on the image, randomly eliminating part of the image information. This aids the model in learning more robust features. Each application of Cutout obscures different parts of the image, aiding in better generalization of the model. An image *I* is defined, with its pixel values represented by matrix *P*, having dimensions W×H×C, where *W* and *H* are the width and height of the image, and *C* is the number of channels. A binary mask *M* represents the Cutout operation, having the same dimensions as *P*, where $M_{i,j,c}\in \left\{0,1\right\}$ indicates whether the channel *c* at position (i,j) is obscured. Having 1 in *M* signifies retaining the pixel value, and 0 means obscuring. The Cutout operation can be represented as

(17) $$I_{\mathrm{cutout}}=I⊙M$$

Here, ⊙ denotes element-wise multiplication. By multiplying the image’s pixel values by the mask, the obscured part of the image $I_{\mathrm{cutout}}$ is obtained.

Cutmix [54] is another method of image data augmentation that differs from Cutout by merging two images, introducing more diversity. The concept of Cutmix is to randomly select an image block and insert it into another image, simultaneously generating a mask corresponding to the inserted image block, thereby achieving image synthesis. Key features of Cutmix include diversity and the introduction of categorical labels. Cutmix introduces more diversity by merging features of different images, aiding the model in learning richer features. It also mixes the categorical labels of the two images, increasing the diversity of labels. Assuming two input images $I_{1}$ and $I_{2}$, their pixel values are represented by matrices $P_{1}$ and $P_{2}$, respectively. There are also two corresponding labels $y_{1}$ and $y_{2}$, representing the categories of these two images. The following equation can generate the Cutmix image $I_{\mathrm{cutmix}}$:

(18) $$I_{\mathrm{cutmix}}=I_{1}⊙M+I_{2}⊙\left(1-M\right)$$

Here, *M* is a mask with the same dimensions as the image, with values between 0 and 1, indicating the selection of the image block. *M* is generated by randomly selecting a rectangular area, setting the corresponding pixel values to 1 and others to 0. This mask can also be used to mix the categorical labels:

(19) $$y_{\mathrm{cutmix}}=\lambda \cdot y_{1}+\left(1-\lambda \right)\cdot y_{2}$$

Here, λ is a randomly generated weight controlling the degree of label mixing.

### 4.3. Proposed Method

#### 4.3.1. Multi-Transformer Overview

In this study, a comprehensive approach based on multimodal and large transformer models was proposed to effectively handle disease detection and question-answering tasks in the agricultural domain. This method integrated advanced multimodal technologies, deep learning, and natural language processing, forming a comprehensive and efficient agricultural intelligence system. Our approach primarily relies on a multi-transformer architecture capable of processing and analyzing data from different modalities (such as images, text). At the core of this architecture is the transformation of various types of data input into a unified format, facilitating effective learning and inference.

Under this framework, data from different modalities are first fused and synchronized through a multimodal alignment module, followed by in-depth analysis of these fused data using a transformer-based inference model, and finally optimizing the overall model performance with a specially designed multimodal loss function. The method flow design includes several key steps. Initially, in the multimodal alignment module, data from various sources are processed and unified. For image data, convolutional neural networks (CNN) are employed to extract features; for text data, NLP techniques are used for word embedding and semantic analysis. Then, features from different modalities are integrated into a unified framework, ensuring effective combination of different types of data in the subsequent processing. Next, the powerful capabilities of the transformer model are utilized in the transformer inference model to process the fused multimodal data. The transformer model, known for its efficient parallel processing and long-distance dependency capturing, excels in handling complex sequential data. In this step, the model not only learns the internal features of the data but also explores the relationships between features from different modalities. Finally, a special multimodal loss function was designed to effectively train this complex system and optimize its performance. This loss function comprehensively considers the characteristics and importance of different modal data and their roles in the final task, ensuring that the model fully considers the characteristics of multimodal data during learning. Theoretically, our method is based on the view that different modalities of data (such as images and text) provide complementary information in agricultural disease detection and question-answering systems. By combining these different data sources, our system can gain a richer and more comprehensive understanding than single modality systems. For example, in disease detection, images provide intuitive disease features, while text offers detailed descriptions and background information about the disease. The combination of this information enables the system to identify and classify diseases more accurately. The adoption of a multi-transformer architecture was due to the advantages of the transformer model in processing sequential data, especially in capturing long-distance dependencies.

#### 4.3.2. Multimodal Alignment Module

**Figure 7:**
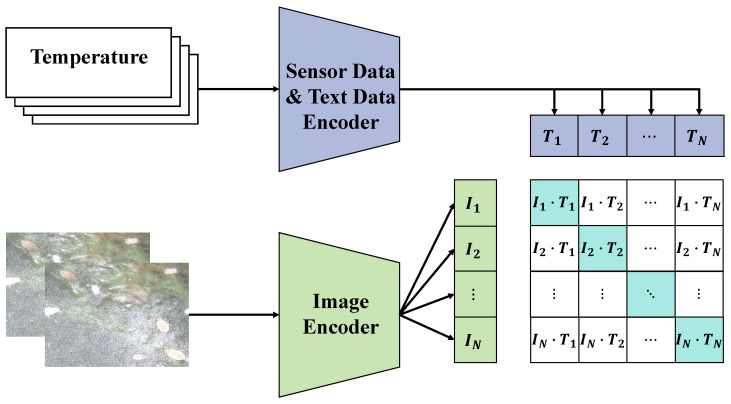
Schematic diagram of a multimodal data processing framework, showing how temperature sensor data and text data are encoded through specific encoders, and how image data are processed through an image encoder. It also illustrates how the encoded data from each source are combined to generate a comprehensive feature representation.

In this study, the multimodal alignment module was one of the core components responsible for effectively fusing data from different modalities (including images, text, and sensor data) to enhance the performance of the agricultural disease detection and question-answering system. The design of the multimodal alignment module aimed to address the differences in feature space and semantic level between the different modal data, providing a unified and coordinated data representation for the subsequent processing and analysis. Inputs to the multimodal alignment module primarily included image and text data. Image data are typically processed by convolutional neural networks (CNN) to extract visual features, while text data are processed using natural language processing technologies (such as BERT) to extract linguistic features. The goal of the multimodal alignment module was to transform these two different modal data features into a unified feature representation for effective integration in the subsequent processing, as shown in Figure 7.

In the processing flow, preliminary feature extraction was first performed on image and text data. For image data *I*, visual features $F_{v}$ were extracted using a CNN model (ResNet50 [23]):

(20) $$F_{v}=CNN\left(I;\theta_{v}\right)$$

Here, $\theta_{v}$ represents the parameters of ResNet50. For text data *T*, linguistic features $F_{t}$ were extracted using a BERT model:

(21) $$F_{t}=BERT\left(T;\theta_{t}\right)$$

Here, $\theta_{t}$ represents the parameters of the BERT model. The key step was feature fusion, where visual features $F_{v}$ and linguistic features $F_{t}$ were combined to generate a unified multimodal feature $F_{m}$. This process could be accomplished using a fusion function *F*:

(22) $$F_{m}=Fusion\left(F_{v},F_{t};\theta_{f}\right)$$

Here, $\theta_{f}$ denotes the parameters of the fusion function. In the multimodal alignment module, the key to feature fusion was finding an effective method to integrate features from different modalities. A weighted fusion method was adopted, where fusion weights were data-driven and learned automatically during model training. Weighted fusion can be represented as

(23) $$F_{m}=\alpha F_{v}+\left(1-\alpha \right)F_{t}$$

Here, α is a learned weight used to balance the importance of features from different modalities. The advantage of this method is its ability to automatically adjust the contribution of visual and linguistic features according to the requirements of different tasks. The application of the multimodal alignment module in the agricultural disease detection and question-answering system brought significant advantages. First, it enabled the system to utilize both visual information from images and semantic information from text, enhancing the accuracy of disease detection and the relevance of question answering. Second, the flexibility of the multimodal alignment module allowed the system to adjust the contribution of data from different modalities according to the characteristics of different tasks, precisely meeting the needs of various tasks. Lastly, this method has a strong generalization capability, adapting to different types and sources of data, enhancing the stability and reliability of the system in practical applications.

#### 4.3.3. Transformer Inference Model

**Figure 8:**
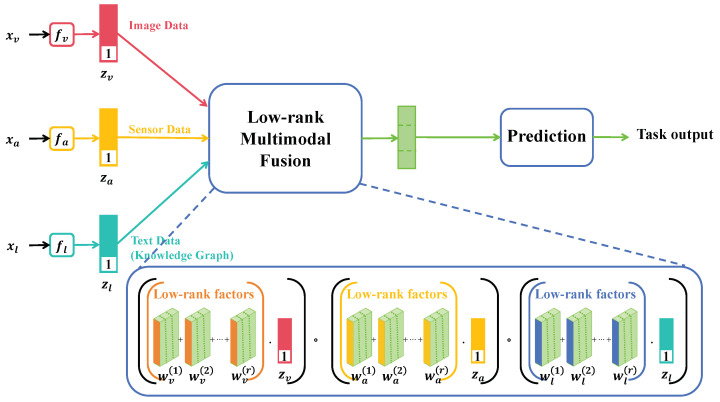
Schematic diagram of a multimodal data low-rank fusion model, depicting how image data, sensor data, and text data (knowledge graphs) are transformed into a low-rank space through specific functional mappings. These low-rank representations are then jointly used for prediction tasks to generate the final task output.

In this research, the transformer inference model, as one of the core components, undertook the critical task of processing and analyzing the fused multimodal data. The transformer model, with its outstanding performance and flexibility, has become the preferred choice in the field of natural language processing for handling complex sequential data. In our study, the transformer model was utilized to extract deep features from fused multimodal data and to conduct effective inference, as shown in Figure 8. The core of the transformer model is its self-attention mechanism, which allows the model to consider all positions in a sequence simultaneously while processing it, thereby capturing complex contextual relationships.

In the inference model of this study, the input is the fused features from the multimodal alignment module. The fused features first pass through a series of transformer encoding layers, each containing a self-attention mechanism and a feed-forward neural network. The working principle of the self-attention mechanism can be represented by the Equation (4). This mechanism enables the model to focus on the associations between different parts of the input sequence. After passing through the self-attention mechanism, the data enter a feed-forward neural network for further processing. The entire process can be represented as

(24) $$Transformer\left(F_{m}\right)=FFN\left(Attention\left(F_{m}\right)\right)$$

Here, $F_{m}$ is the output from the multimodal alignment module, and FFN denotes the feed-forward neural network. The transformer model has significant advantages in processing sequential data. In particular, its self-attention mechanism can effectively handle long-distance dependency issues, which is crucial for understanding and analyzing complex multimodal data. Additionally, the parallel processing capability of the transformer model makes it more efficient in handling large-scale data. Mathematically, the advantage of the transformer model lies in its self-attention mechanism’s ability to dynamically weight different parts of the sequence. By adjusting weights, the model can more flexibly capture important features in the sequence, thereby improving the accuracy of inference. The application of the transformer inference model in the agricultural disease detection and question-answering system brought several advantages: The transformer model can extract rich and deep features from fused multimodal data, crucial for understanding complex agricultural issues. In processing long sequential data such as descriptive text and image labels, the Transformer model can effectively capture long-distance dependencies. The parallel processing ability of the Transformer model makes it more efficient in handling a large amount of multimodal data, which is particularly important for building practical agricultural intelligence systems.

#### 4.3.4. Multimodal Loss Function

In this research, a specialized multimodal loss function has been designed to optimize and evaluate the transformer inference model based on multimodal data. This multimodal loss function takes into consideration the characteristics of different modal data and their significance in the model, ensuring optimal learning outcomes when the model processes multimodal data. The design of the multimodal loss function acknowledges the distinct roles played by different modal data in the model within multimodal learning tasks. By introducing modality-specific loss functions, the model is guaranteed to fully consider the characteristics of each modality during learning, thereby enhancing its capability to handle multimodal data. The design principle of the multimodal loss function is based on the notion that different modalities contribute differently to the model, and these contributions may vary with the task. For instance, in some scenarios, image data might provide more intuitive information than text data, while in others, semantic information from a text may be more crucial. Therefore, our loss function design aimed to dynamically balance these different modal contributions to enhance the overall performance of the model. The multimodal loss function combined traditional classification loss (such as cross-entropy loss) with modality-specific loss. Its mathematical expression can be represented as

(25) $$L_{total}=\alpha L_{classification}+\beta L_{modal1}+\gamma L_{modal2}$$

Here, $L_{total}$ denotes the total loss, $L_{classification}$ is the cross-entropy loss for classification tasks, and $L_{modal1}$ and $L_{modal2}$ represent losses related to different modalities (for example, specific loss for image and specific loss for text). α, β, and γ are weight coefficients used to balance the loss from different parts. Cross-entropy loss is a common loss function in classification tasks and used to measure the difference between the probability distribution predicted by the model and the actual label distribution. Its mathematical formula is as follows:

(26) $$L_{classification}=-\sum_{i}y_{i}\log\left(p_{i}\right)$$

Here, $y_{i}$ is the actual label’s probability distribution, and $p_{i}$ is the model’s predicted probability distribution. For modality-specific losses, different loss functions can be designed according to the task. For instance, for the image modality, loss functions related to image reconstruction or feature matching might be used; for the text modality, loss functions related to semantic similarity or sentence generation quality might be employed. The application of the multimodal loss function in agricultural disease detection and question-answering system offers several advantages. By balancing the contributions of different modal data, the multimodal loss function can improve the model’s accuracy in processing multimodal data. Different tasks may require varying degrees of attention to different modal data. The design of the multimodal loss function allows the model to automatically adjust the importance of different modal data based on the task’s characteristics. By combining classification loss and modality-specific loss, the multimodal loss function can optimize the model performance.

### 4.4. Experimental Configuration

#### 4.4.1. Hardware Platform

The hardware platform forms the foundation for deep learning experiments and is crucial for research on multimodal disease detection and agricultural question-answering systems. This section details the configuration of the hardware platform, including GPUs, CPUs, memory, and other aspects. In our hardware platform, an NVIDIA GeForce RTX 3090 was selected as the primary GPU. This GPU, based on NVIDIA’s Ampere architecture, boasts numerous CUDA cores and substantial memory capacity, making it well-suited for processing multimodal data and large-scale models. On the other hand, the CPU (central processing unit) plays a significant role in data preprocessing, model deployment, and certain computation-intensive tasks. A server equipped with a 32-core CPU was chosen for our hardware platform. This CPU, with multiple physical and logical cores, is capable of handling multi-threaded tasks and supports high-performance computing. In multimodal tasks, datasets are often large, requiring ample memory for data loading and processing. Hence, 128 GB of RAM was configured to ensure sufficient memory for model training and inference. Large-scale datasets necessitate high-speed storage devices to accelerate data loading and saving. Therefore, a high-performance solid state drive (SSD) was chosen as the primary storage device to provide rapid data access.

#### 4.4.2. Software Configuration and Hyperparameter Settings

In deep learning research, appropriate software configuration and hyperparameter settings are vital for training models in multimodal disease detection and agricultural question-answering systems. This section details the software configuration and various hyperparameter settings, including the deep learning framework, operating system, learning rate, batch size, and more. In multimodal tasks, choosing the right deep learning framework is critical for model training and performance. One of the current most popular deep learning frameworks is PyTorch, known for its extensive library support, dynamic computation graph, and user-friendly API. PyTorch was selected as the main deep learning framework for its excellent performance in multimodal tasks and substantial community support. Selecting an appropriate operating system is also a crucial decision. Linux operating systems are widely used in deep learning research and development, due to their good support for deep learning tools and libraries. In our experiments, a popular Linux distribution, Ubuntu, was chosen to ensure compatibility with deep learning tools. The learning rate is a key hyperparameter in deep learning that determines the step size of the model during each parameter update. The choice of learning rate directly affects the model’s convergence speed and performance. An initial learning rate of 0.001 was used in our experiments. Different learning rate settings were tried, and the best one was chosen based on the performance on the validation set. Batch size refers to the number of training samples input to the model at once. Batch training helps speed up the training process and improve memory efficiency. The choice of batch size depends on the model’s architecture and hardware resources. Larger batch sizes can accelerate training but also require more memory. With limited hardware resources, a smaller batch size may need to be chosen. Therefore, in this case, a batch size of 128 was set. Adjustments and optimizations to batch size were made during experiments to achieve optimal performance. To prevent model overfitting, regularization techniques, including L2 regularization and dropout, were applied. Regularization helped the model generalize to new data. Additionally, an appropriate optimizer, Adam, was chosen to update the model parameters. Model parameter initialization is also an important aspect. We used pretrained model weights for initialization. Pretrained models, usually trained on large-scale datasets, have better initial feature representations. For the multimodal tasks, we chose pretrained text and image models and combined them into a multimodal model. Hyperparameter search methods were used in our experiments to find the best combination of hyperparameters, including searching for the best learning rate, batch size, regularization parameters, etc. Techniques such as grid search, random search, and Bayesian optimization were employed to find the best hyperparameter settings. Hyperparameter search is an iterative process, requiring repeated trials of different hyperparameter combinations, guided by the performance on the validation set.

#### 4.4.3. Dataset Training

In deep learning tasks, appropriately partitioning the dataset for training, validation, and testing is of paramount importance. The methods of dataset splitting and cross-validation directly impact the performance assessment and generalizability of the model. In this paper, the details of dataset splitting, K-Fold cross-validation, and other training-related aspects are discussed. Dataset splitting is one of the primary steps in machine learning experiments. An appropriate method of dataset splitting ensures that the model can fully utilize the data during training, validation, and testing processes. In the tasks of multimodal disease detection and agricultural question-answering systems, there is a comprehensive dataset containing a large amount of data, which need to be divided into three key parts. The training set, constituting 70% of the total dataset, is the foundation for model training, wherein the model learns to capture patterns and features of the data. The validation set, making up 15% of the total dataset, is used for hyperparameter tuning and performance evaluation of the model. Multiple validations are conducted on the validation set to choose the optimal model hyperparameter settings, such as learning rate and regularization parameters. The test set, comprising the remaining 15% of the dataset, is utilized for the final evaluation of the model’s performance. The performance assessment of the model on the test set serves as the ultimate metric to measure the model’s performance on real-world data. When splitting the dataset, it is crucial to ensure that each part contains data from different categories or samples, to guarantee the generalizability of the model. Random sampling is employed for splitting to maintain an even distribution of data. Additionally, K-Fold (k = 10) cross-validation is used, allowing for fuller use of data and providing reliable performance evaluation. This approach involves dividing the dataset into K equally sized subsets, where K − 1 subsets are used for training and the remaining one for validation. This process is repeated K times, with a different subset serving as the validation set each time, and the average of the K validation scores is taken as the final performance metric. The benefits of K-Fold cross-validation include obtaining a more accurate performance estimate through multiple validations, reducing the impact of randomness, and the ability to try different hyperparameter settings on each validation fold to select the best settings.

#### 4.4.4. Model Evaluation Metrics

To assess the effectiveness of our disease detection and agricultural question-answering system, we relied on three principal metrics for evaluation.

Accuracy is a metric frequently utilized in classification tasks. It quantifies the percentage of samples that the model has classified correctly out of the total number of samples. In simpler terms, accuracy can be described as the ratio of the count of samples correctly identified by the model to the overall count of samples examined. Precision measures the accuracy of the model in identifying positive samples. Specifically, it calculates the percentage of samples that were accurately predicted as positive from the pool of all samples that the model labeled as positive. This means that precision is determined by dividing the number of true positive samples (those correctly identified as positive) by the number of all samples that the model predicted as positive. Recall, also known as sensitivity, focuses on the model’s ability to correctly identify all possible positive samples. It represents the fraction of positive samples that were correctly predicted as positive out of the total actual positive samples. To put this another way, recall is the quotient obtained when the number of true positive samples is divided by the total number of samples that are actually positive.

These metrics served to gauge the model’s accuracy in identifying diseases and providing answers to agricultural queries. Accuracy provided a broad view of the model’s overall performance, while precision and recall offered insights into its effectiveness in scenarios where the distribution of data might have been skewed.

## 5. Conclusions

In this study, a comprehensive approach based on multimodal data and the transformer model was proposed to address key challenges in agricultural disease detection and question-answering systems. First, in the disease detection experiments, various models including AlexNet, GoogLeNet, VGG, ResNet, and the method proposed in this paper were compared. The results demonstrated that the proposed method achieved the highest values in precision, recall, and accuracy, with respective scores of 0.95, 0.92, and 0.94, significantly outperforming the other comparative models. This indicated that the proposed method has a significant advantage in identifying various agricultural diseases, particularly in processing complex data and subtle features. Second, in the agricultural image captioning experiment, the performance of BLIP, mPLUG-Owl, InstructBLIP, CLIP, BLIP2, and the method proposed in this paper was examined. In this task, the proposed method also displayed the best performance, with a precision, recall, and accuracy scores of 0.92, 0.88, and 0.91, respectively. These results suggest that the proposed method can effectively understand the content of agricultural images and generate accurate and rich descriptive texts, which is important for enhancing the level of intelligence and automation in agricultural production. In the object detection experiment, SSD, RetinaNet, CenterNet, YOLOv8, and the method proposed in this paper were compared. The experimental results showed that the proposed method performed best in terms of precision, recall, and accuracy, achieving scores of 0.96, 0.91, and 0.94 respectively. This result reaffirms the efficiency and accuracy of the proposed method in processing complex agricultural data, especially in accurately identifying and locating agricultural diseases. Additionally, multimodal dataset ablation experiments and different loss function ablation experiments were conducted. In the multimodal dataset ablation experiment, it was found that the model performed optimally when using full modal data (image, text, and sensor data), and the absence of any modality led to a decrease in performance. This emphasized the importance of multimodal data in enhancing model performance. In the different loss function ablation experiments, it was found that the multimodal loss function performed best in all tasks, proving its effectiveness in handling multimodal data.

## Figures and Tables

**Table 1:** Comparison of disease detection performance.

Model	Precision	Recall	Accuracy
AlexNet	0.83	0.81	0.82
GoogLeNet	0.86	0.84	0.85
VGG	0.89	0.87	0.88
ResNet	0.92	0.90	0.91
Proposed Method	0.95	0.92	0.94

**Table 2:** Comparison of performance in agricultural image captioning.

Model	Precision	Recall	Accuracy
BLIP	0.78	0.74	0.75
mPLUG-Owl	0.80	0.76	0.77
InstructBLIP	0.84	0.80	0.82
CLIP	0.86	0.82	0.85
BLIP2	0.89	0.85	0.88
Proposed Method	0.92	0.88	0.91

**Table 3:** Comparison of object detection performance.

Model	Precision	Recall	Accuracy
SSD	0.82	0.80	0.81
RetinaNet	0.85	0.83	0.84
CenterNet	0.89	0.87	0.88
YOLOv8	0.93	0.90	0.92
Proposed Method	0.96	0.91	0.94

**Table 4:** Object detection result details for our method.

Crop	Disease	Precision	Recall	Accuracy
Rice	Rice Blast	0.97	0.92	0.95
	Sheath Blight	0.95	0.93	0.94
	Rice False Smut	0.92	0.90	0.91
	Bacterial Leaf Blight	0.87	0.85	0.86
	Downy Mildew	0.97	0.94	0.96
Wheat	Rust	0.98	0.93	0.95
	Powdery Mildew	0.96	0.94	0.95
	Fusarium Head Blight	0.95	0.91	0.93
	Loose Smut	0.78	0.76	0.77
	Sheath Blight	0.97	0.92	0.94
Potato	Early Blight	0.96	0.91	0.93
	Late Blight	0.95	0.92	0.94
	Leafroll Disease	0.94	0.90	0.92
	Wilt Disease	0.96	0.94	0.95
	Black Scurf	0.97	0.93	0.95
Cotton	Wilt Disease	0.95	0.92	0.94
	Yellow Wilt	0.93	0.90	0.92
	Verticillium Wilt	0.96	0.94	0.95
	Blight	0.94	0.91	0.93
	Anthracnose	0.97	0.95	0.96
Corn	Rust	0.95	0.93	0.94
	Northern Corn Leaf Blight	0.96	0.92	0.94
	Common Smut	0.97	0.94	0.95
	Southern Corn Leaf Blight	0.74	0.70	0.72
	Leaf Spot Disease	0.98	0.96	0.97

**Table 5:** Multimodal dataset ablation experiment.

Image Data	Text Data	Sensor Data	Precision	Recall	Accuracy
✓	✓	✓	0.96	0.93	0.94
✗	✗	✓	0.24	0.21	0.23
✗	✓	✗	0.78	0.73	0.75
✓	✗	✗	0.92	0.90	0.91

**Table 6:** Different loss function ablation experiment.

Task	Loss Function	Precision	Recall	Accuracy
Disease Detection	Hinge Loss	0.90	0.85	0.86
	MSE Loss	0.93	0.87	0.91
	Multimodal Loss	0.95	0.92	0.94
Agricultural Image Captioning	Hinge Loss	0.84	0.79	0.82
	MSE Loss	0.89	0.83	0.86
	Multimodal Loss	0.92	0.8	0.89
Object Detection	Hinge Loss	0.88	0.84	0.85
	MSE Loss	0.91	0.87	0.89
	Multimodal Loss	0.96	0.92	0.94

**Table 7:** Image dataset details.

Crop	Disease	Number
Rice	Rice Blast	768
	Sheath Blight	1095
	Rice False Smut	677
	Bacterial Leaf Blight	1135
	Downy Mildew	983
Wheat	Rust	690
	Powdery Mildew	734
	Fusarium Head Blight	918
	Loose Smut	1129
	Sheath Blight	885
Potato	Early Blight	921
	Late Blight	1079
	Leafroll Disease	776
	Wilt Disease	698
	Black Scurf	993
Cotton	Wilt Disease	874
	Yellow Wilt	903
	Verticillium Wilt	1005
	Blight	1297
	Anthracnose	793
Corn	Rust	754
	Northern Corn Leaf Blight	913
	Common Smut	952
	Southern Corn Leaf Blight	1045
	Leaf Spot Disease	1176

## Data Availability

The data presented in this study are available on request from the corresponding author.

## References

[1] Zhang Y., Wa S., Sun P., Wang Y. (2021). Pear defect detection method based on resnet and dcgan. Information.

[2] Saleem M.H., Potgieter J., Arif K.M. (2021). Automation in agriculture by machine and deep learning techniques: A review of recent developments. Precis. Agric..

[3] Sujatha R., Chatterjee J.M., Jhanjhi N., Brohi S.N. (2021). Performance of deep learning vs machine learning in plant leaf disease detection. Microprocess. Microsyst..

[4] Zhang Y., Wa S., Liu Y., Zhou X., Sun P., Ma Q. (2021). High-accuracy detection of maize leaf diseases CNN based on multi-pathway activation function module. Remote Sens..

[5] Li L., Zhang S., Wang B. (2021). Plant disease detection and classification by deep learning—A review. IEEE Access.

[6] Ray M., Ray A., Dash S., Mishra A., Achary K.G., Nayak S., Singh S. (2017). Fungal disease detection in plants: Traditional assays, novel diagnostic techniques and biosensors. Biosens. Bioelectron..

[7] Vadamalai G., Kong L.L., Iftikhar Y. (2020). Plant Genetics and Physiology in Disease Prognosis. Plant Disease Management Strategies for Sustainable Agriculture through Traditional and Modern Approaches.

[8] Das D., Singh M., Mohanty S.S., Chakravarty S. Leaf disease detection using support vector machine. Proceedings of the 2020 International Conference on Communication and Signal Processing (ICCSP).

[9] Lin X., Wa S., Zhang Y., Ma Q. (2022). A dilated segmentation network with the morphological correction method in farming area image Series. Remote Sens..

[10] Zhang Y., Yang X., Liu Y., Zhou J., Huang Y., Li J., Zhang L., Ma Q. (2024). A time-series neural network for pig feeding behavior recognition and dangerous detection from videos. Comput. Electron. Agric..

[11] Deepalakshmi P., Lavanya K., Srinivasu P.N. (2021). Plant leaf disease detection using CNN algorithm. Int. J. Inf. Syst. Model. Des. (IJISMD).

[12] Sharma P., Berwal Y.P.S., Ghai W. (2020). Performance analysis of deep learning CNN models for disease detection in plants using image segmentation. Inf. Process. Agric..

[13] Bedi P., Gole P. (2021). Plant disease detection using hybrid model based on convolutional autoencoder and convolutional neural network. Artif. Intell. Agric..

[14] De Silva M., Brown D. (2023). Multispectral Plant Disease Detection with Vision Transformer–Convolutional Neural Network Hybrid Approaches. Sensors.

[15] Parez S., Dilshad N., Alghamdi N.S., Alanazi T.M., Lee J.W. (2023). Visual intelligence in precision agriculture: Exploring plant disease detection via efficient vision transformers. Sensors.

[16] Thai H.T., Le K.H., Nguyen N.L.T. (2023). FormerLeaf: An efficient vision transformer for Cassava Leaf Disease detection. Comput. Electron. Agric..

[17] Xie L., Yuille A. Genetic cnn. Proceedings of the IEEE International Conference on Computer Vision.

[18] Redmon J., Divvala S., Girshick R., Farhadi A. You only look once: Unified, real-time object detection. Proceedings of the IEEE Conference on Computer Vision and Pattern Recognition.

[19] Hu Z., Dong Y., Wang K., Chang K.W., Sun Y. Gpt-gnn: Generative pre-training of graph neural networks. Proceedings of the 26th ACM SIGKDD International Conference on Knowledge Discovery & Data Mining.

[20] Devlin J., Chang M.W., Lee K., Toutanova K. (2018). Bert: Pre-training of deep bidirectional transformers for language understanding. arXiv.

[21] Trong V.H., Gwang-hyun Y., Vu D.T., Jin-young K. (2020). Late fusion of multimodal deep neural networks for weeds classification. Comput. Electron. Agric..

[22] Simonyan K., Zisserman A. (2014). Very deep convolutional networks for large-scale image recognition. arXiv.

[23] He K., Zhang X., Ren S., Sun J. Deep residual learning for image recognition. Proceedings of the IEEE Conference on Computer Vision and Pattern Recognition.

[24] Singh S., Ahuja U., Kumar M., Kumar K., Sachdeva M. (2021). Face mask detection using YOLOv3 and faster R-CNN models: COVID-19 environment. Multimed. Tools Appl..

[25] Wu W., Liu H., Li L., Long Y., Wang X., Wang Z., Li J., Chang Y. (2021). Application of local fully Convolutional Neural Network combined with YOLO v5 algorithm in small target detection of remote sensing image. PloS ONE.

[26] Bertasius G., Wang H., Torresani L. Is space-time attention all you need for video understanding?. Proceedings of the 38th International Conference on Machine Learning.

[27] Patil R.R., Kumar S. (2022). Rice-fusion: A multimodality data fusion framework for rice disease diagnosis. IEEE Access.

[28] Dandrifosse S., Carlier A., Dumont B., Mercatoris B. (2021). Registration and fusion of close-range multimodal wheat images in field conditions. Remote Sens..

[29] Anandhi D.R.F.R., Sathiamoorthy S. (2023). Enhanced Sea Horse Optimization with Deep Learning-based Multimodal Fusion Technique for Rice Plant Disease Segmentation and Classification. Eng. Technol. Appl. Sci. Res..

[30] Gadiraju K.K., Ramachandra B., Chen Z., Vatsavai R.R. Multimodal deep learning based crop classification using multispectral and multitemporal satellite imagery. Proceedings of the 26th ACM SIGKDD International Conference on Knowledge Discovery & Data Mining.

[31] Qing J., Deng X., Lan Y., Li Z. (2023). GPT-aided diagnosis on agricultural image based on a new light YOLOPC. Comput. Electron. Agric..

[32] Cao Y., Sun Z., Li L., Mo W. (2022). A study of sentiment analysis algorithms for agricultural product reviews based on improved bert model. Symmetry.

[33] Zhang Y., Lv C. (2024). TinySegformer: A lightweight visual segmentation model for real-time agricultural pest detection. Comput. Electron. Agric..

[34] Vaswani A., Shazeer N., Parmar N., Uszkoreit J., Jones L., Gomez A.N., Kaiser Ł., Polosukhin I. (2017). Attention is all you need. Adv. Neural Inf. Process. Syst..

[35] Shen Y., Wang L., Jin Y. AAFormer: A multi-modal transformer network for aerial agricultural images. Proceedings of the IEEE/CVF Conference on Computer Vision and Pattern Recognition.

[36] Fountas S., Espejo-Garcia B., Kasimati A., Mylonas N., Darra N. (2020). The future of digital agriculture: Technologies and opportunities. IT Prof..

[37] Lippi M., Bonucci N., Carpio R.F., Contarini M., Speranza S., Gasparri A. A yolo-based pest detection system for precision agriculture. Proceedings of the 2021 29th Mediterranean Conference on Control and Automation (MED).

[38] Lu J., Tan L., Jiang H. (2021). Review on convolutional neural network (CNN) applied to plant leaf disease classification. Agriculture.

[39] Zhang Y., Yang G., Liu Y., Wang C., Yin Y. (2022). An improved YOLO network for unopened cotton boll detection in the field. J. Intell. Fuzzy Syst..

[40] Krizhevsky A., Sutskever I., Hinton G.E. Imagenet classification with deep convolutional neural networks. Proceedings of the Advances in Neural Information Processing Systems.

[41] Szegedy C., Liu W., Jia Y., Sermanet P., Reed S., Anguelov D., Erhan D., Vanhoucke V., Rabinovich A. Going deeper with convolutions. Proceedings of the IEEE Conference on Computer Vision and Pattern Recognition.

[42] Li J., Li D., Xiong C., Hoi S. Blip: Bootstrapping language-image pre-training for unified vision-language understanding and generation. Proceedings of the International Conference on Machine Learning.

[43] Ye Q., Xu H., Xu G., Ye J., Yan M., Zhou Y., Wang J., Hu A., Shi P., Shi Y. (2023). mplug-owl: Modularization empowers large language models with multimodality. arXiv.

[44] Dai W., Li J., Li D., Tiong A., Zhao J., Wang W., Li B., Fung P., Hoi S. (2023). InstructBLIP: Towards General-purpose Vision-Language Models with Instruction Tuning. arXiv.

[45] Radford A., Kim J.W., Hallacy C., Ramesh A., Goh G., Agarwal S., Sastry G., Askell A., Mishkin P., Clark J. Learning transferable visual models from natural language supervision. Proceedings of the International Conference on Machine Learning. PMLR.

[46] Li J., Li D., Savarese S., Hoi S. (2023). Blip-2: Bootstrapping language-image pre-training with frozen image encoders and large language models. arXiv.

[47] Liu W., Anguelov D., Erhan D., Szegedy C., Reed S., Fu C.Y., Berg A.C. Ssd: Single shot multibox detector. Proceedings of the Computer Vision–ECCV 2016: 14th European Conference.

[48] Lin T.Y., Goyal P., Girshick R., He K., Dollár P. Focal loss for dense object detection. Proceedings of the IEEE International Conference on Computer Vision.

[49] Zhou X., Wang D., Krähenbühl P. (2019). Objects as points. arXiv.

[50] Zhang L., Ding G., Li C., Li D. (2023). DCF-Yolov8: An Improved Algorithm for Aggregating Low-Level Features to Detect Agricultural Pests and Diseases. Agronomy.

[51] Zhang Y., Wang Y. (2022). High-precision wheat head detection model based on one-stage network and GAN model. Front. Plant Sci..

[52] Bender A., Whelan B., Sukkarieh S. (2020). A high-resolution, multimodal data set for agricultural robotics: A Ladybird’s-eye view of Brassica. J. Field Robot..

[53] DeVries T., Taylor G.W. (2017). Improved regularization of convolutional neural networks with cutout. arXiv.

[54] Yun S., Han D., Oh S.J., Chun S., Choe J., Yoo Y. Cutmix: Regularization strategy to train strong classifiers with localizable features. Proceedings of the IEEE/CVF International Conference on Computer Vision.

